# Dengue-1 Envelope Protein Domain III along with PELC and CpG Oligodeoxynucleotides Synergistically Enhances Immune Responses

**DOI:** 10.1371/journal.pntd.0001645

**Published:** 2012-05-15

**Authors:** Chen-Yi Chiang, Ming-Hsi Huang, Chun-Hsiang Hsieh, Mei-Yu Chen, Hsueh-Hung Liu, Jy-Ping Tsai, Yi-Shiuan Li, Ching-Yun Chang, Shih-Jen Liu, Pele Chong, Chih-Hsiang Leng, Hsin-Wei Chen

**Affiliations:** 1 National Institute of Infectious Diseases and Vaccinology, National Health Research Institutes, Miaoli, Taiwan; 2 Graduate Institute of Immunology, China Medical University, Taichung, Taiwan; Florida Gulf Coast University, United States of America

## Abstract

The major weaknesses of subunit vaccines are their low immunogenicity and poor efficacy. Adjuvants can help to overcome some of these inherent defects with subunit vaccines. Here, we evaluated the efficacy of the newly developed water-in-oil-in-water multiphase emulsion system, termed PELC, in potentiating the protective capacity of dengue-1 envelope protein domain III. Unlike aluminum phosphate, dengue-1 envelope protein domain III formulated with PELC plus CpG oligodeoxynucleotides induced neutralizing antibodies against dengue-1 virus and increased the splenocyte secretion of IFN-γ after in vitro re-stimulation. The induced antibodies contained both the IgG1 and IgG2a subclasses. A rapid anamnestic neutralizing antibody response against a live dengue virus challenge was elicited at week 26 after the first immunization. These results demonstrate that PELC plus CpG oligodeoxynucleotides broaden the dengue-1 envelope protein domain III-specific immune responses. PELC plus CpG oligodeoxynucleotides is a promising adjuvant for recombinant protein based vaccination against dengue virus.

## Author Summary

Dengue is a mosquito-borne disease. Infection of dengue virus can cause clinical manifestations ranging from self-limiting dengue fever to potentially life-threatening dengue hemorrhagic fever or dengue shock syndrome. In recent years, dengue has spread to most tropical and subtropical areas, making it a global health concern. Specific approaches for dengue therapy do not exist; the development of a dengue vaccine would represent a major advance in the control of the disease. Currently, no licensed dengue vaccine is available. Subunit vaccines provide a great safety strategy for developing dengue vaccine. However, the major weaknesses of subunit vaccines are low immunogenicity and poor efficacy. Here we employed dengue-1 envelope protein domain III as a model vaccine candidate and described a newly developed water-in-oil-in water multiphase emulsion system to overcome the inherent defect of subunit vaccines. We showed that emulsification of dengue-1 envelope protein domain III and CpG oligodeoxynucleotides synergistically broadened immune responses and potentiated the protective capacity of dengue-1 envelope protein domain III. These results provide valuable information for development of recombinant protein based vaccination against dengue virus and future clinical studies.

## Introduction

Dengue is the most important mosquito-borne flavivirus disease. People living in the tropical and subtropical areas are at risk of dengue virus infection, and more than 50 million dengue infected cases occur worldwide each year [1], [2]. Vaccine inoculation is a cost-effective way of combating the threat of infectious diseases. In the past six decades, tremendous effort has been made to develop a dengue vaccine [3]–[5]. However, despite these efforts, no licensed dengue vaccines are currently available.

Many advanced biological technologies have been applied to dengue vaccine development, and numbers of vaccine approaches are currently in pre-clinical or clinical development. These approaches include chimerization with other flaviviruses or the deletion of portions of the genomes to obtain live attenuated dengue vaccines, viral vector vaccines, DNA vaccines, and recombinant subunit vaccines [3]–[5]. All of the approaches are associated with different advantages and disadvantages. Among these approaches, the recombinant subunit vaccine provides the greatest degree of safety.

Dengue envelope protein domain III has been shown to be involved in host receptor binding [6], [7], and several neutralizing epitopes have been identified within this domain [8]–[13]. These characteristics of the envelope protein domain III indicate that it would be a promising dengue vaccine candidate [14]. Several subunit vaccines based on recombinant dengue envelope protein domain III have been developed to protect against dengue viral infection [15]–[23]. Formulating dengue subunit vaccine candidates with proper adjuvants [15]–[17], [21]–[23] or expressing vaccine candidates in a lipoprotein [18]–[20] was necessary to enhance their immunogenicity. These results indicate that one of the major weaknesses of subunit vaccines is their low immunogenicity and that appropriate adjuvants or delivery systems are required to overcome this weakness.

Adjuvants and delivery systems have noticeably improved over the past several years. We previously developed a bioresorbable diblock tri-component copolymer poly(ethylene glycol)-block-poly(lactide-co-ε- caprolactone) mixed with squalene and Span®85 to produce homogeneous nano-particles (PELC). This water-in-oil-in-water multiphase emulsion system can be utilized for vaccine delivery [24]–[26]. In addition, we demonstrated that a formulation of inactivated influenza virus and CpG oligodeoxynucleotides (CpG) could enhance both the overall immune response and cross-clade protective immunity [27]. These results indicate that PELC-formulated vaccines has improved potential efficacy.

In this study, we evaluated the potential of aluminum phosphate, CpG, PELC, and PELC plus CpG as adjuvants to enhance the immunogenicity of recombinant dengue-1 envelope protein domain III (D1ED III). We demonstrated that recombinant D1ED III formulated with PELC plus CpG induced stronger and broader immune responses than using other adjuvant formulations. These results provide valuable information for future clinical studies.

## Materials and Methods

### Ethics statement

Animal studies were carried out in strict accordance with the recommendations from Taiwan's Animal Protection Act. The protocol was approved by the Animal Committee of the National Health Research Institutes (Protocol No: NHRI-IACUC-098014) and were performed according to their guidelines.

### Cloning and expression of recombinant D1ED III

A consensus sequence for D1ED III from dengue-1 viruses was obtained by aligning five amino acid sequences from different isolates of the dengue-1 virus [21]. According to the amino acid sequence of D1ED III, the DNA sequence of the D1ED III gene was derived using codon usage of *Escherichia coli* and was fully synthesized using the assembly PCR method [28]. The product of the assembly PCR was then amplified by conventional PCR. To generate an expressing plasmid for recombinant D1ED III, the following primers were used: forward primer, 5′-GGAATTCCATATGaaaggcatgagctatgtgatgt -3′ (Nde I site, underlined); reverse primer, 5′- CCGCTCGAGgctgctgccttttttaaa -3′ (Xho I site, underlined). The PCR product was cloned into the expression vector pET-22b(+) (Novagen, Madison, WI), using Nde I and Xho I sites to produce the pDen1E3 plasmid. As a result, the C-terminus of the recombinant protein contained an additional hexahistidine tag (HisTag). The *Escherichia coli* strain BL21 (DE3) (Invitrogen, Carlsbad, CA) was transformed with the expression plasmid pDen1E3 for protein expression. The transformed cells were cultured at 37°C overnight, and protein expression was induced by adding 1 mM isopropylthiogalactoside for 20 hours at 20°C.

### Purification and characterization of recombinant D1ED III

The recombinant D1ED III was purified by disrupting the harvested cells in a French Press (Constant Systems, Daventry, UK) at 27 Kpsi in homogenization buffer (20 mM Tris (pH 8.0), 50 mM sucrose, 500 mM NaCl and 10% glycerol). The cell lysate was clarified by centrifugation (80,000× *g* for 40 min). The majority of recombinant D1ED III was found in the soluble fraction. The recombinant D1ED III was purified using immobilized metal affinity chromatography (IMAC) columns. The eluent from the IMAC column was then polished using an anion exchange column (Q sepharose fast flow; GE) after dialysis against Q buffer (20 mM Tris-Cl, 1 m MEDTA, pH 8.0). E membrane (Pall, USA) was used to remove the endotoxin. The endotoxin levels of the purified recombinant D1ED III were determined by the Limulus amebocyte lysate assay (Associates of Cape Cod, Inc. Cape Cod, MA), and the resulting endotoxin levels were found to be below the detection limit of the kit (<3 EU/mg). The fractions from each step were analyzed by SDS-PAGE gel stained with Coomassie blue (Coomassie Brilliant Blue R-250) and were immunoblotted with anti-HisTag antibodies.

The purified recombinant D1ED III was dialyzed against 5 mM ammonium bicarbonate, pH 8.5. Dialyzed samples were mixed with trypsin (Promega Co., Madison, WI, USA) at a 50∶1 ratio (wt/wt) in 25 mM ammonium bicarbonate, pH 8.5. The reaction allowed to continue for 2 hours and stopped by adding formic acid at a final concentration of 1.2%. The tryptic peptides were analyzed by MALDI-TOF (Burker) mass spectrometry.

### Adjuvant preparation

Murine CpG was synthesized by Invitrogen Taiwan Ltd and was given as a 10 µg dose dissolved in the sterile water or in the antigenic media. The CpG sequence used was 5′-TCCATGACGTTCCTGACGTT-3′ with all phosphorothioate backbones. Aluminum phosphate suspension was kindly provided by the Taiwan CDC and given as a 300 µg dose in acidic media (pH 6). PELC is a squalene W/O/W nanoemulsion stabilized by Span®85 (sorbitan trioleate, Sigma-Aldrich, Steinheim, Germany) and PEG-*b*-PLACL, the latter consisting of 75 wt-% of hydrophilic bioabsorbable PEG and 25 wt-% of lipophilic biodegradable PLACL with molecular weight of 7,000 daltons as previously described [24]–[26]. Briefly, 120 mg of PEG-*b*-PLACL, 0.8 mL of phosphate buffer saline (PBS), and 1.1 mL of oily solution consisting of squalene (Sigma-Aldrich, Steinheim, Germany) and Span®85 (85/15 v/v) were emulsified using Polytron®PT 3100 homogenizer (Kinematica AG, Switzerland) at 6,000 rpm for 5 min. The emulsified PELC formulation was stored at 4°C until use. PELC-formulated vaccine was investigated by re-dispersing 0.2 mL of stock emulsion into 1.8 mL of aqueous solution and mixed with a test-tube rotator (Labinco LD-79, Netherlands) at 5 rpm for at least 1 hour before injection. Recombinant D1ED III and/or CpG were introduced in the aqueous solution, respectively.

### Virus

Dengue-1 (Hawaii) was used for this study. The virus was propagated in Vero cells, and viral titers were determined by focus-forming assays with BHK-21 cells.

### Mouse experiments

Five BALB/c mice (6–8 weeks of age) were immunized subcutaneously with recombinant D1ED III (10 µg per dose, unless indicated). Mice were given one or two immunizations at a two-week interval with the same regimen. To detect the anamnestic response generated by immunization, immunized mice were inoculated intraperitoneally with 3×10^6^ focus-forming units (FFU) of live dengue-1 virus. Blood was collected from each mouse at different time points, as indicated. Sera were prepared and stored at −20°C until use.

### ELISPOT assays

The numbers of IFN-γ- and IL-4-producing cells were determined using mouse IFN-γ and IL-4 ELISPOT kits (eBioscience), respectively. All of the assays were performed according to the manufacturer's instructions. Briefly, 96-well plates with PVDF membranes (Millipore) were coated with capture antibody and incubated at 4°C for 18 hours. The plates were washed twice and blocked with RPMI medium supplemented with fetal bovine serum (10%) for one hour to prevent nonspecific binding in later steps. Splenocytes were seeded at a concentration of 5×10^5^ cells/well and stimulated with D1ED III (10 µg/mL) for 3 days at 37°C in a 5% CO_2_ humidified incubator. After incubation, the cells were removed from the plates by washing three times with 0.05% (w/v) Tween 20 in PBS. A 100 µL aliquot of biotinylated detection antibody was then added to each well. The plates were incubated at 37°C for 2 hours. The washing steps were repeated as above, and after a 45-minute incubation at room temperature with the avidin-horseradish peroxidase (HRP) complex reagent, the plates were washed three times with 0.05% (w/v) Tween 20 in PBS and then three times with PBS alone. A 100 µL aliquot of 3-amine-9-ethyl carbazole (Sigma-Aldrich) staining solution was added to each well to develop the spots. The reaction was stopped after one hour by placing the plates under tap water. The spots were counted using an ELISPOT reader (Cellular Technology Ltd.). The values presented in the results are mean ± standard deviation of each group.

### Antibody response measurement

The levels of anti-D1ED III IgG in the serum samples were determined by titrating the samples. Sera were diluted using 3-fold serial dilutions (starting at 1∶33). Briefly, purified D1ED III was coated on 96-well plates. In some experiments, supernatant obtained from dengue-1 virus infected Vero cells was coated on 96-well plates (2×10^4^ ffu virus/well). Bound IgG was detected with HRP-conjugated goat anti-mouse IgG Fc. After the addition of 3, 3′, 5, 5′-tetramethylbenzidine (TMB), the absorbance was measured with an ELISA reader at 450 nm. For measurement of IgG1 and IgG2a anti-D1ED III subclass, biotin-conjugated rat anti-mouse IgG1 and rat anti-mouse IgG2a were used as detectors, and avidin-HRP was then added. Color was developed as described above. ELISA end-point titers were defined as the serum dilution that gave a 0.5 OD value. The serum dilution was obtained from the titration curve by interpolation, unless the OD value was less than 0.5 at the starting dilution (1∶33).

### Immunofluorescence assay

BHK-21 cells were infected with dengue-1 virus. Three days after infection, the cells were fixed for 15 min in 3.7% formaldehyde/PBS. After washing with PBS, the cells were permeabilized with 0.1% Nonidet P40/PBS for 15 min and blocked with 3% bovine serum albumin (BSA)/PBS for 30 min. Viruses in the infected cells were detected by mouse pre-immune and immune sera (from D1ED III-immunized mice). After washing with PBS, antibody-labeled cells were detected using a secondary antibody conjugated with fluorescein isothiocyanate (FITC). Cellular DNA was labeled by Hoechst stains.

### Focus reduction neutralization tests (FRNT)

Sera were diluted using 2-fold serial dilutions (starting at 1∶8) and the sera were heat inactivated prior to testing. A monolayer of BHK-21 cells in 24-well plates was inoculated with dengue-1 virus that had been pre-mixed at 4°C overnight with pre-immunization or post-immunization sera to a final volume of 0.5 mL. The virus titer prior to pre-mixing was about 20–40 FFU per well. Viral adsorption was allowed to proceed for 3 hours at 37°C. An overlay medium containing 2% fetal bovine serum and 0.8% methylcellulose in DMEM was added at the conclusion of adsorption. The infected monolayer was incubated at 37°C. After 72 hours of infection, the overlay medium was removed from the wells, and the BHK cells were washed with cold PBS. The cells were fixed for 15 min in 3.7% formaldehyde/PBS. After washing with PBS, the cells were permeabilized with 0.1% Nonidet P40/PBS for 15 min and blocked with 3% bovine serum albumin (BSA)/PBS for 30 min. Infected cells were detected by a monoclonal anti-dengue antibody (American Type Culture Collection, No. HB-114). After washing with PBS, antibody-labeled cells were detected using a secondary antibody conjugated to HRP. The labeling was visualized using TMB. The FFUs were counted, and the neutralizing antibody titer FRNT_50_ (or FRNT_80_) was calculated as the reciprocal of the highest dilution that produced a 50% (or 80%) reduction of FFU compared with control samples containing the virus alone. For calculation purpose, the neutralizing antibody titer was designated as 2^2^ when neutralizing antibody titer was less than 2^3^.

### Blocking of dengue virus infection to BHK-21 cells by D1ED III

To test whether D1ED III blocks dengue virus infection of BHK-21 cells, virus was pre-mixed with different amount D1ED III or control BSA protein as indicated for 10 min at 4°C. Viral adsorption was allowed to proceed for 3 hours at 37°C. The FFUs were determined as described above.

### Statistical analyses

The statistical analysis was conducted using GraphPad Prism version 5.02 (GraphPad Software, Inc.). Data from D1ED III blocking dengue-1 viral infection were processed by a two-tailed Student's *t*-test. Data from ELISPOT assay were analyzed by the Mann-Whitney test. Data from ELISA and FRNT were performed by the ANOVA Bonferroni post test. Differences with a *p* value of less than 0.05 were considered statistically significant.

## Results

### Expression and purification of recombinant dengue-1 envelope protein domain III

**Figure 1:**
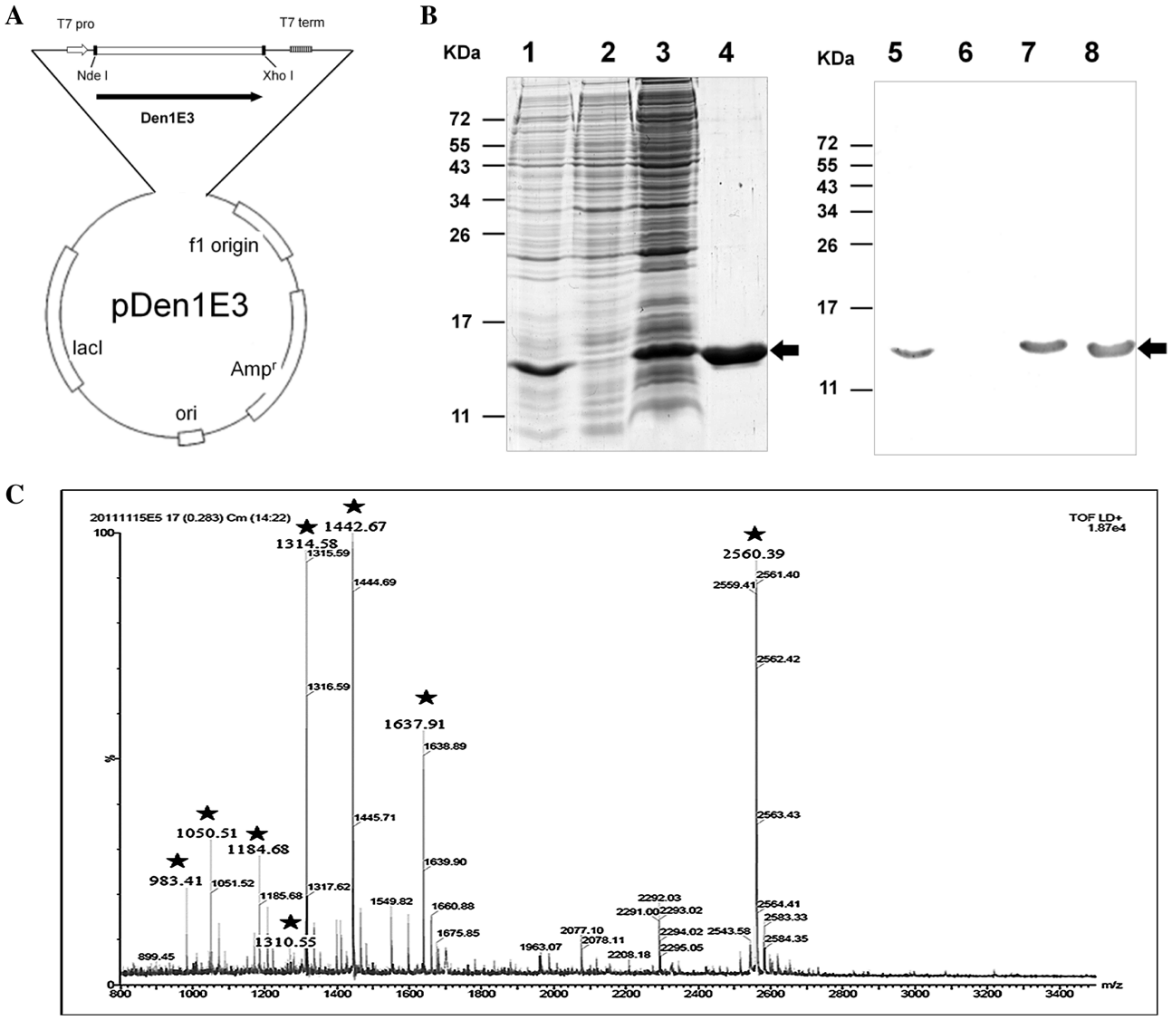
Expression and purification of recombinant D1ED III. (A) The DNA sequence of the D1ED III gene was derived using codon usage of *Escherichia coli* and fully synthesized using the assembly PCR method. The PCR product was cloned into the pET22b vector for recombinant D1ED III expression. The recombinant protein contained an additional HHHHHH sequence (HisTag) at the C-terminus and was expressed under the control of the T7 promoter. (B)The recombinant D1ED III protein purification process was monitored using 17.5% reducing SDS-PAGE followed stained by Coomassie Blue staining and immunoblotting using anti-HisTag antibodies. The recombinant D1ED III was expressed in *Escherichia coli* strain BL21 (DE3). Lane 1, recombinant D1ED III expression after IPTG induction; lane 2, protein expression in the absence of IPTG induction; lane 3, soluble fraction of recombinant D1ED III; lane 4, purified recombinant D1ED III. Lanes 5–8 show immunoblotting to monitor the recombinant D1ED III induction and purification processes, and the samples in these lanes are the same as those in lanes 1–4, respectively. The arrows indicate the electrophoretic positions of recombinant D1ED III in the gels or blots. (C) Recombinant D1ED III was digested with trypsin to assess their peptide mass fingerprinting. The results confirmed that the major peaks in the mass spectra were derived from recombinant D1ED III.

The D1ED III amino acid sequence is a consensus sequence of dengue virus type 1 aligned from selected target sequences (Accession number: P27909, P27913, P17763, P33478 and P27912) [21]. The DNA sequence of the D1ED III gene was derived using codon usage with *Escherichia coli* and was fully synthesized using the assembly PCR method. The PCR product was cloned into the pET22b vector for recombinant D1ED III expression. The recombinant protein contained an additional HHHHHH sequence (HisTag) at the C-terminus and was expressed under the control of the T7 promoter (Figure 1A). The recombinant D1ED III was purified using an immobilized metal affinity chromatography (IMAC) column and an anion exchange column (Figure 1B, lanes 1–4). Recombinant D1ED III was detected with anti-HisTag antibodies (Figure 1B, lanes 5–8). After the lipopolysaccharide (LPS) was removed (less than 3 EU/mg), purified recombinant D1ED III was comparatively analyzed for its immunogenicity and efficacy in animal models. Recombinant D1ED III was also digested with trypsin to assess its peptide mass fingerprinting. The molecular weight of recombinant D1ED III is 12377. All major peaks in the spectrum at m/z 983.41, 1050.51, 1184.68, 1310.55, 1314.58, 1442.68, 1637.88, and 2559.29 cover over 80% of recombinant D1ED III. The results confirmed that the major peaks in the mass spectra were derived from recombinant D1ED III (Figure 1C).

### Recombinant D1ED III can block dengue-1 viral infection

**Figure 2:**
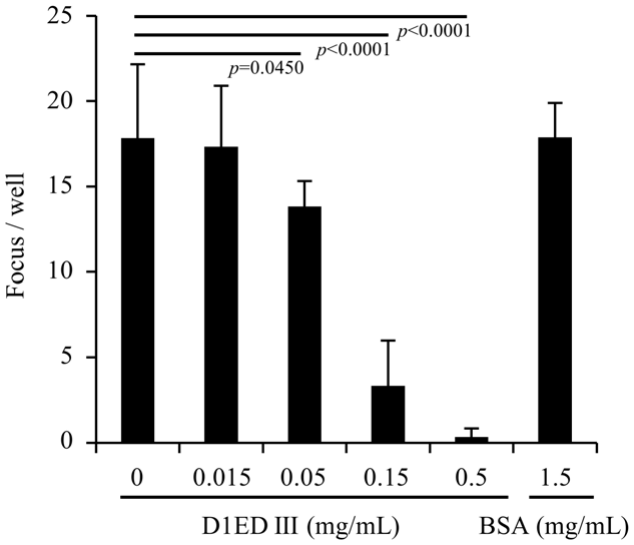
Soluble D1ED III inhibits dengue-1 infection to BHK 21 cells. Dengue-1 virus was pre-mixed with different amount of D1ED III or control bovine serum albumin (BSA) protein as indicated for 10 min at 4°C. Viral adsorption on a monolayer of BHK-21 cells in 24-well plates proceeded for 3 hours at 37°C. The FFUs were determined 3 days after the viral infection. The results are pooled from two independent experiments. The *p* values were calculated using an unpaired two-tailed Student's *t*-test.

Dengue envelope protein domain III has been show to be involved in cellular receptor binding [6], [7]. We hypothesized that if purified recombinant D1ED III exists in a suitable conformation, then soluble D1ED III should interfere with dengue viral infections. As shown in Figure 2, the ability of dengue-1 virus to infect BHK-21 cells was inhibited in the presence of D1ED III in a dosage-dependent manner. Greater than 80% reduction of focus number was observed when D1ED III added to cells at a concentration of 0.15 mg/mL. BSA did not inhibit dengue-1 focus formation at concentrations as high as 1.5 mg/mL, which is 10-fold higher than D1ED III. These results suggest that D1ED III can block the cellular binding sites of the dengue-1 virus.

### Immunogenicity of purified D1ED III formulated with different adjuvants

**Figure 3:**
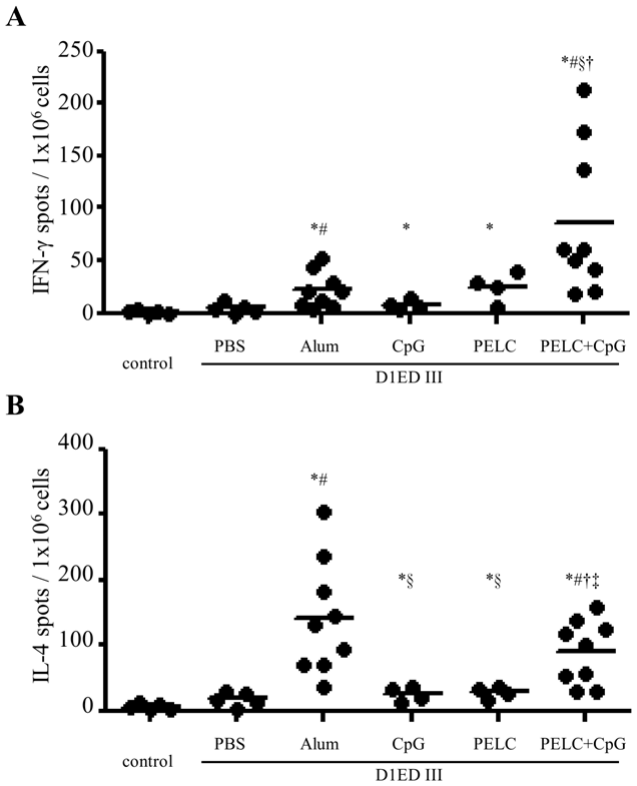
Cytokine profiles induced by D1ED III formulated with various adjuvants. BALB/c mice were immunized subcutaneously twice with 10 µg of D1ED III in PBS or in combination with Alum, CpG, PELC, or PELC plus CpG at a two-week interval. Mice immunized with PBS alone (without D1ED III) served as controls. Seven days after the final immunization, splenocytes were stimulated with D1ED III (10 µg/mL) for 72 h. The frequencies of (A) IFN-γ- and (B) IL-4-secreting cells in the spleens were determined using mouse IFN-γ and IL-4 ELISPOT kits, respectively. The results are pooled from two independent experiments. The *p* values were calculated using the two-tailed Mann-Whitney test. ^*^ *p*<0.05 compared to control. ^#^ *p*<0.05 compared to PBS. ^§^ *p*<0.05 compared to Alum. ^†^ *p*<0.05 compared to CpG. ^‡^ *p*<0.05 compared to PELC.

The purified recombinant D1ED III vaccine candidate was formulated with different adjuvants and then tested for its ability to induce both T- and B-cell immune responses in mice. Groups of BALB/c mice were immunized with the different formulations two times with a two-week interval between immunizations. Animals immunized with PBS alone served as controls. One week after the second immunization, splenocytes were harvested and examined for IFN-γ and IL-4 secretion in response to three days of D1ED III stimulation. The ability of the different formulations to induce cytokine secretion varied greatly, as shown in Figure 3. The frequency of IFN-γ spots per 10^6^ splenocytes in mice immunized with recombinant D1ED III alone was 4.9±4.4 (n = 5) spots. Splenocytes from mice immunized with recombinant D1ED III formulated with aluminum phosphate (21.6±17.4, n = 9, *p*<0.05 compared to D1ED III alone), CpG (7.6±4.3, n = 4, *p*>0.05 compared to D1ED III alone), and PELC (24.9±14.3, n = 4, *p*>0.05 compared to D1ED III alone) showed modest increases in IFN-γ spots. Mice immunized with recombinant D1ED III formulated with PELC plus CpG elicited the highest number of IFN-γ spots (85.9±70.3, n = 9, *p*<0.05 compared to D1ED III alone, alum, or CpG) (Figure 3A). Although the formulation using PELC plus CpG elicited fewer IL-4 spots than the aluminum phosphate formulation (89.7±48.6 vs. 140.7±87.0), the difference was not statistically significant (*p* = 0.1903). Interestingly, the PELC plus CpG formulation elicited more IL-4 spots than the formulations using PELC (28.3±9.7, *p*<0.05) or CpG (25.1±10.9, *p*<0.05) alone (Figure 3B).

**Figure 4:**
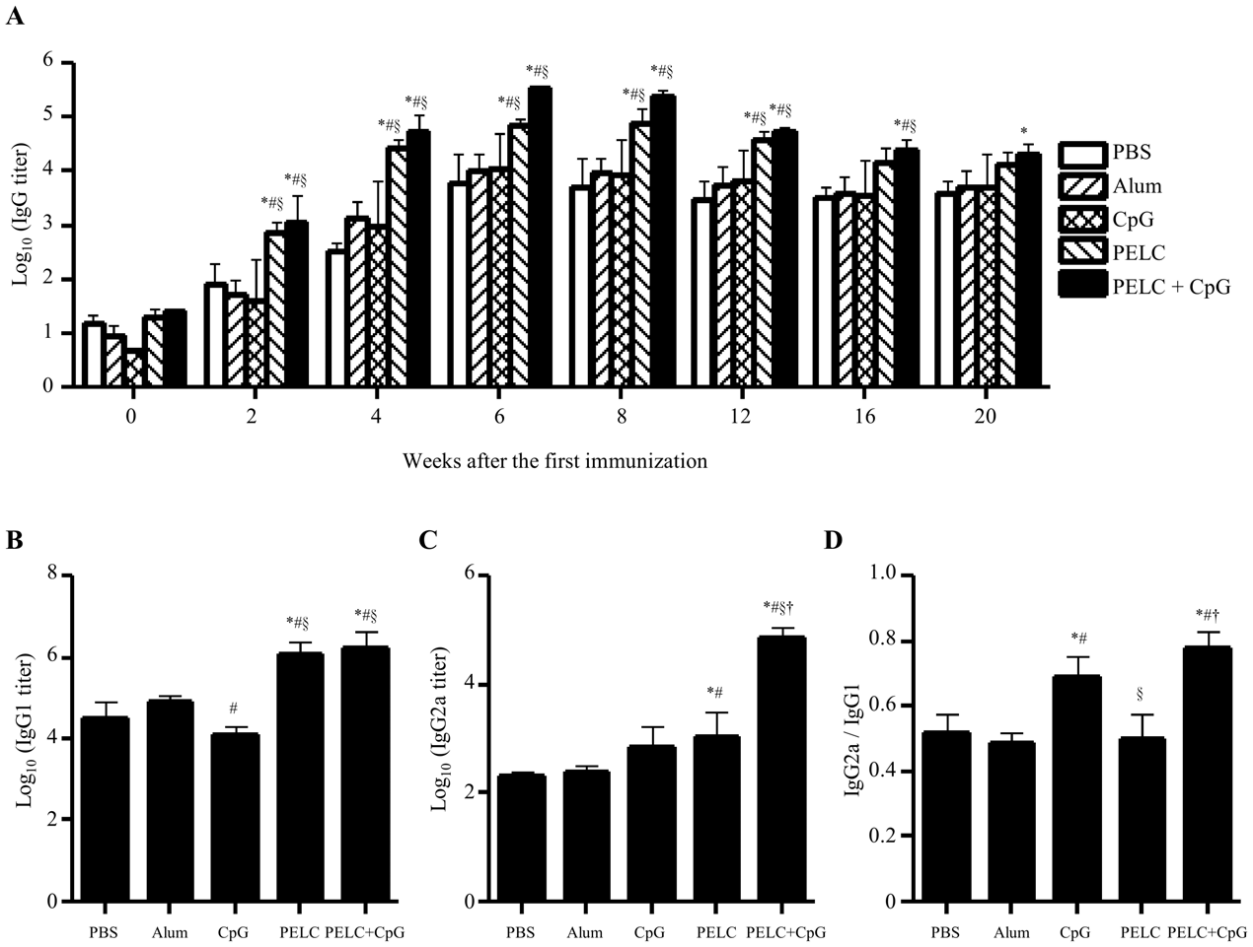
Humoral immune responses induced by D1ED III formulated with various adjuvants. Groups of BALB/c mice (n = 5) were immunized subcutaneously with 10 µg of D1ED III three times either in PBS or in combination with Alum, CpG, PELC, or PELC plus CpG at a two-week interval. (A) Sera were collected from mice at the indicated time points after the first immunization. IgG antibodies against D1ED III were evaluated using ELISA. Pre-immune sera (week 0) were collected and used as basal levels for comparison. (B) IgG1 and (C) IgG2a antibodies of serum samples obtained from 6 weeks post-prime immunization against D1ED III were evaluated by ELISA. (D) The ratios of IgG2a/IgG1 are shown. ELISA end-point titers were defined as the serum dilution that gave a 0.5 OD value. The serum dilution was obtained from the titration curve by interpolation, unless the OD value was less 0.5 at the starting dilution (1∶33). The results are expressed as the mean ± SD. Statistical significance was determined by the ANOVA Bonferroni post test at the same time point. ^*^ *p*<0.05 compared to PBS. ^#^ *p*<0.05 compared to Alum. ^§^ *p*<0.05 compared to CpG. ^†^ *p*<0.05 compared to PELC.

Next, we evaluated the IgG antibody responses following three immunizations with two-week intervals between immunizations. Serum samples were collected from the immunized mice at different time points, as indicated in Figure 4A. Formulations of recombinant D1ED III with PELC or PELC plus CpG were highly immunogenic and generated stronger antibody responses than the other formulation groups (*p*<0.05 by the ANOVA Bonferroni post test). All of the antibody responses peaked between week 4 and week 8 after the first immunization. Importantly, substantial levels of anti-D1ED III IgG antibodies were detectable for over 20 weeks after the initial priming. Sera collected from different formulation groups at week 6 were analyzed for presence of IgG1 (Figure 4B) and IgG2a (Figure 4C). Mice immunized with the CpG formulation generated lower levels of IgG1 antibody than mice that received the aluminum phosphate, PELC, and PELC plus CpG formulations (*p*<0.05 by the ANOVA Bonferroni post test). The PELC and PELC plus CpG groups had significant levels of IgG1 in comparison with the other groups (*p*<0.05 by the ANOVA Bonferroni post test). The combination of PELC and CpG induced the highest levels of IgG2a (*p*<0.05 by the ANOVA Bonferroni post test). As shown in Figure 4D, the ratios of IgG2a to IgG1 in mice receiving PELC plus CpG formulation were higher than those observed in mice receiving the antigen alone, aluminum phosphate, or PELC formulations (*p*<0.05 by the ANOVA Bonferroni post test). These results indicate that combination of CpG and PELC could improve the IgG2a response.

### D1ED III induced antibodies that recognize and neutralize the infectivity of the dengue-1 virus

**Figure 5:**
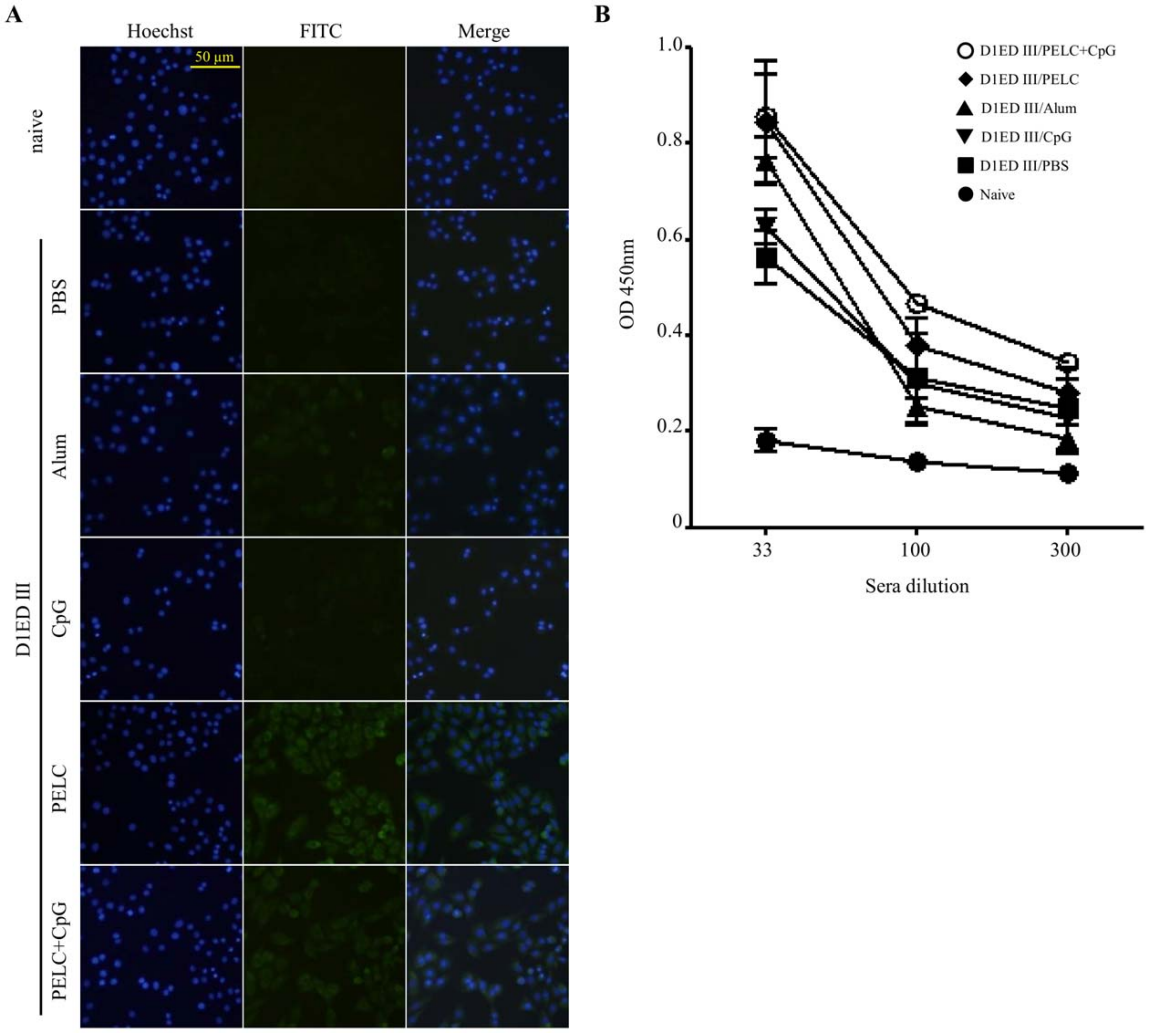
The capability of D1ED III induced antibodies to recognize and neutralize dengue-1 virus. Mice were immunized with D1ED III (10 µg/dose) formulated with the indicated adjuvants three times at two-week intervals. (A) Serum samples were collected 8 weeks after the first immunization. BHK-21 cells were infected with dengue-1 virus at multiplicity of infection 0.3 for 3 days. Virus-infected cells were fixed and probed with sera drawn from before (naïve) or after immunization with D1ED III. Cellular DNA was visualized by Hoechst stains (left panels). Dengue-1 virus infected cells were shown as FITC stains (middle panels). The right panels show the images of merging Hoechst and FITC stains. Magnification for each image is 200×. (B) Serum samples were collected 8 weeks after the first immunization. ELISA was performed by using dengue-1 virus as coating antigen. Naïve sera were used as basal levels for comparison. The results are presented as mean ± SD from triplicate wells.

As the preceding experiments showed that the various formulations of recombinant D1ED III used could elicit considerable antibody responses, we wondered whether these antibodies would recognize the dengue-1 virus. To address this, we employed indirect immunofluorescence staining to evaluate antibody specificity in the sera of mice immunized with the various formulations of recombinant D1ED III. As shown in Figure 5A, naïve serum did not produce immunofluorescent reactivity with dengue-1 virus infected cells. Weak immunofluorescence signals were observed in sera obtained from mice immunized with recombinant D1ED III alone or formulated with CpG. In contrast, strong immunofluorescence signals were observed in sera obtained from mice immunized with the aluminum phosphate, PELC, and PLEC plus CpG formulations. These results suggest that the antibodies induced by recombinant D1ED III can react with dengue-1 virus.

To further examine whether D1ED III raised antibodies can recognize native dengue-1 virus, ELISA was performed by using dengue-1 virus coated 96-well plates. In comparison with naïve serum, sera obtained from mice immunized with D1ED III alone or with various adjuvants produced significantly higher OD values (*p*<0.05 by the ANOVA Bonferroni post test). These results demonstrated that antibodies induced by D1ED III can recognize native dengue-1 virus (Figure 5B).

The major objective of this study was to explore whether any of the formulations of recombinant D1ED III could induce neutralizing antibodies responses. To evaluate the dengue-1 virus neutralizing ability of the antibodies induced by vaccination, antisera from the mouse immunized with the various formulations were collected, and the neutralizing antibody titers were assessed by focus reduction neutralization tests (FRNT). As shown in Table 1, mice immunized with recombinant D1ED III formulated without adjuvant or with aluminum phosphate, CpG or PELC could not generate significant neutralizing antibody responses (neutralizing antibody titers FRNT_50_<2^3^). In contrast, mice received PELC plus CpG formulation elicited detectable neutralizing antibody titers (FRNT_50_ = 2^4.6^).

**Table 1:** Neutralizing antibody titers in mice immunized with D1ED III formulated with various adjuvants^1^.

Adjuvants	FRNT_50_					FRNT_80_				
	Mouse 1	2	3	4	5	1	2	3	4	5
PBS	<8	<8	<8	8	8	<8	<8	<8	<8	<8
Alum	<8	<8	8	8	16	<8	<8	<8	<8	<8
CpG	<8	<8	<8	<8	<8	<8	<8	<8	<8	<8
PELC	<8	<8	8	8	8	<8	<8	<8	<8	<8
PELC+CpG	8	16	16	64	64* # § † 2	<8	<8	<8	<8	8

1 Mice were immunized with D1ED III (10 µg/dose) formulated with the indicated adjuvants three times at two-week intervals. Serum samples (n = 5) were collected on 2 weeks after the last immunization. The dengue-1 virus neutralizing capacity was determined by FRNT. The neutralizing antibody titer was calculated as the reciprocal of the highest dilution that resulted in a 50% or 80% reduction of FFU compared to control samples containing the virus alone.

2 Statistical analysis was performed by the ANOVA Bonferroni post test.

*: *p*<0.05 compared to PBS.

# *p*<0.05 compared to Alum.

**§:** *p*<0.05 compared to CpG.

**†:** *p*<0.05 compared to PELC.

### Formulation of D1ED III with PELC plus CpG can induce anamnestic neutralizing antibody responses

As the formulation of recombinant D1ED III with PELC plus CpG could elicit the strongest cellular and humoral immune responses and neutralizing antibody responses of all the formulations we tested, we further evaluated the protective efficacy of the D1ED III with PELC plus CpG formulation. Groups of BALB/c mice were immunized with various amounts (3, 10, or 30 µg/dose) of D1ED III three times at two-week intervals. The animals were then injected intraperitoneally with dengue-1 virus (3×10^6^ FFU/mouse) 26 weeks after the first immunization. Serum samples were collected from the mice at the indicated time points, and the neutralizing capacity against dengue-1 virus was examined. As shown in Table 2, no significant neutralizing activity was detected in the sera obtained from naïve mice before and after viral infection (neutralizing antibody titers <2^3^). Mice immunized with 3, 10, or 30 µg of D1ED III produced neutralizing antibody responses at week 6 after initial priming. The neutralizing antibody titers waned at week 24 after the first immunization. At 6 days post-viral challenge, the neutralizing antibody titers FRNT_50_ were 2^3.8^, 2^4.4^, and 2^4.2^ in mice received vaccination with 3, 10, or 30 µg/dose, respectively. These results provide tangible evidence that an efficient anamnestic neutralizing antibody response was induced in mice immunized with recombinant D1ED III formulated with PELC plus CpG.

**Table 2:** Neutralizing antibody titers in mice immunized with D1ED III formulated with PELC plus CpG before and after viral challenge^1^.

Time	Groups	FRNT_50_					FRNT_80_				
		Mouse 1	2	3	4	5	1	2	3	4	5
6 weeks post prime	Naïve	<8	<8	<8	8	8	<8	<8	<8	<8	<8
	3 µg	8	8	16	16	32	<8	<8	8	8	8
	10 µg	8	16	32	32	32* 2	<8	<8	8	8	16*
	30 µg	8	8	16	32	64*	8	8	8	16	32* # §
24 weeks post prime	naïve	<8	<8	<8	<8	<8	<8	<8	<8	<8	<8
	3 µg	<8	<8	<8	8	16	<8	<8	<8	<8	<8
	10 µg	<8	<8	<8	<8	<8	<8	<8	<8	<8	<8
	30 µg	<8	<8	<8	<8	<8	<8	<8	<8	<8	<8
6 days post challenge	naïve	<8	<8	<8	8	8	<8	<8	<8	<8	<8
	3 µg	8	8	16	16	32*	<8	<8	<8	<8	8
	10 µg	8	16	32	32	32*	<8	<8	<8	8	8
	30 µg	8	8	16	32	64*	<8	8	8	8	8*
14 days post challenge	naïve	<8	<8	<8	<8	<8	<8	<8	<8	<8	<8
	3 µg	<8	<8	<8	8	32	<8	<8	<8	<8	<8
	10 µg	<8	8	8	8	32	<8	<8	<8	<8	<8
	30 µg	<8	8	16	32	32*	<8	<8	<8	<8	<8
22 days post challenge	naïve	<8	<8	<8	<8	8	<8	<8	<8	<8	<8
	3 µg	<8	<8	<8	<8	8	<8	<8	<8	<8	<8
	10 µg	<8	<8	8	8	32	<8	<8	<8	<8	<8
	30 µg	<8	16	32	32	32* #	<8	<8	<8	<8	8

1 Groups of BALB/c mice (n = 5) were immunized three times subcutaneously with 3, 10, or 30 µg of D1ED III formulated with PELC plus CpG at two-week intervals. Naïve mice without immunization served as controls. All animals were inoculated intraperitoneally with 3×10^6^ FFU of live dengue-1 virus at week 26 post-priming. Sera were collected at different time points as indicated. The sera's ability to neutralize dengue-1 virus was determined by FRNT. The neutralizing antibody titer was calculated as the reciprocal of the highest dilution that resulted in a 50% or 80% reduction of FFU compared to control samples containing the virus alone.

2 Statistical analysis was performed by the ANOVA Bonferroni post test at the same time point.

*: *p*<0.05 compared to naive.

# *p*<0.05 compared to 3 µg.

**§:** *p*<0.05 compared to 10 µg.

## Discussion

Adjuvants containing aluminum (alum) are currently the most widely used adjuvants in human vaccines. However, formulations of dengue subunit vaccines using alum were unable to induce complete protection against dengue virus infection [15], [29]–[32]. In the present study, we prepared D1ED III as a dengue subunit vaccine candidate (Figure 1). The purified D1ED III formed in the proper conformation, which could occupy the cellular binding sites to reduce dengue virus infection (Figure 2). We also found that D1ED III formulated with aluminum phosphate could not induce a significant neutralizing antibody response (Table 1). Altogether, these results suggest that alum may not be suitable for dengue subunit vaccines. In the present study, we evaluated the suitability of PELC-based adjuvants to potentiate the neutralizing antibody capacity of the D1ED III in the mouse model.

All the mice immunized with D1ED III formulated without or with various adjuvants could induce a D1ED III-specific antibody response (Figure 4A). We also noticed that antibodies elicited in all of the immunized groups could recognize dengue-1 virus infected cells (Figure 5A and 5B). However, none of antibodies exhibited a significant neutralizing capacity aside from the antibodies obtained from PELC plus CpG immunized mice (Table 1). In addition, sizeable anamnestic neutralizing antibody responses were observed in mice immunized with D1ED III formulated with PELC plus CpG (Table 2). These results indicate that PELC plus CpG is a promising potential adjuvant for dengue subunit vaccines.

The antibody-dependent enhancement (ADE) of flavivirus infection can be inhibited by complement protein C1q. The inhibition effect is IgG subclass-specific. ADE induced by an IgG2a monoclonal antibody but not by an epitope-matched IgG1 monoclonal antibody could be inhibited by purified C1q [33]. Our results show that different adjuvant formulations can alter the ratio of IgG1/IgG2a (Figure 4D). Interestingly, a significant level of IgG2a was induced in mice immunized with the PELC plus CpG formulation (Figure 4C). These results indicate that D1ED III formulated with PELC plus CpG induces an IgG2a-biased response that may possibly reduce the risk of ADE when sufficient serum C1q levels are present.

Alum predominantly induces a Th2 polarized response [34]–[37] featuring IL-4 production. Consistent with these findings, D1ED III formulated with aluminum phosphate induced higher IL-4 production than any of the other adjuvant formulations that we tested (Figure 3B). Interestingly, the PELC plus CpG formulation induced both vigorous IFN-γ and IL-4 responses (Figure 3). IFN-γ has been shown to play an important role in antiviral activity against dengue virus [38], [39]. The induction of strong IFN-γ responses by the PELC plus CpG formulation will be greatly advantageous in protecting hosts against dengue virus.

There are some of limitations for in vivo protection studies due to the lack of a relevant mouse model of dengue infection. In the present study, we utilized dengue-1 virus as an antigenic challenge model to evaluate memory neutralizing antibody responses. Our results show that the low neutralizing antibody titers were induced and diminished at 24 weeks after immunization of D1ED III formulated with PELC plus CpG (Table 2). Interestingly, quick anamnestic neutralizing antibody responses were evoked when stimulated with dengue-1 virus in mice immunized with D1ED III formulated with PELC plus CpG but not naïve mice (Table 2). These results suggest that memory neutralizing antibody responses were induced in mice immunized with D1ED III formulated with PELC plus CpG.

Our findings show that the PELC plus CpG formulation improves both the intensity and quality of the immune responses against D1ED III. Moreover, the immune responses induced by the PELC plus CpG formulation are beneficial to host protection against dengue viral infection. In conclusion, PELC plus CpG is an attractive adjuvant for dengue subunit vaccines based on recombinant envelope protein domain III. Future work should expand to test the suitability of PELC plus CpG formulations in non-human primate studies.
